# *tert*-Butyl *N*-[1-diazo­acetyl-3-(methyl­sulfan­yl)prop­yl]carbamate

**DOI:** 10.1107/S1600536809030815

**Published:** 2009-08-08

**Authors:** Tahir Mehmood, Javid H. Zaidi, Peter G. Jones

**Affiliations:** a Department of Chemistry, Quaid-I-Azam University, Islamabad 45320, Pakistan; b Institut für Anorganische und Analytische Chemie, Technische Universität Braunschweig, Hagenring 30, 38106 Braunschweig, Germany

## Abstract

In the enanti­omerically pure title compound, C_11_H_19_N_3_O_3_S, the chain C—N—C(O)—O—C—C (from the asymmetric carbon to a methyl of the *tert*-butyl group) displays an extended conformation. In the crystal, mol­ecules are linked into chains parallel to the *c* axis by classical N—H⋯O_diazo­carbon­yl_ hydrogen bonding and an unusual inter­molecular three-centre inter­action involving the amino acid (aa) carbonyl O_aa_ and the diazo­carbonyl grouping C(O)—CH—N N, with H⋯O_aa_ = 2.51 Å and N⋯O_aa_ = 2.8141 (14) Å.

## Related literature

For the applications of α-diazo­carbonyl compounds in organic and, especially, natural product synthesis, see: Padwa & Weingarten (1996 ▶). The ready availability, relative stability and facile decomposition of these compounds under various conditions make them useful inter­mediates, see: Doyle *et al.* (1998 ▶). α-Diazo­ketones undergo a variety of transformations, see: Ye & McKervey (1994 ▶). Asymmetric versions of diazo­carbonyl reactions have been reported to produce enanti­omerically pure compounds, see: Doyle & McKervey (1997 ▶). The Arndt-Eistert synthesis, which consists of conversion of activated carboxylic acids to diazo­ketones by the action of diazo­methane followed by Wolf rearrangement, has become widely used in recent years for the synthesis of β-peptides and β-amino acid derivatives from appropriately protected α-amino acids, see: Müller *et al.* (1998 ▶).
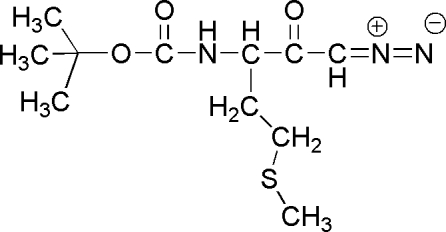


## Experimental

### 

#### Crystal data

C_11_H_19_N_3_O_3_S
*M* *_r_* = 273.35
Trigonal, 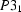

*a* = 9.7915 (3) Å
*c* = 13.8581 (5) Å
*V* = 1150.62 (6) Å^3^
*Z* = 3
Cu *K*α radiation
μ = 1.93 mm^−1^
*T* = 100 K
0.20 × 0.20 × 0.15 mm

#### Data collection

Oxford Diffraction Xcalibur Nova A diffractometer
Absorption correction: multi-scan (CrysAlis Pro; Oxford Diffraction, 2009 ▶) *T*_min_ = 0.717, *T*_max_ = 1.000 (expected range = 0.537–0.749)
16152 measured reflections
3073 independent reflections
3051 reflections with *I* > 2σ(*I*)
*R*_int_ = 0.032

#### Refinement

*R*[*F* ^2^ > 2σ(*F* ^2^)] = 0.023
*wR*(*F* ^2^) = 0.060
*S* = 1.05
3073 reflections
171 parameters
1 restraint
H atoms treated by a mixture of independent and constrained refinement
Δρ_max_ = 0.15 e Å^−3^
Δρ_min_ = −0.14 e Å^−3^
Absolute structure: Flack (1983 ▶), 1474 Freidel pairs
Flack parameter: 0.023 (10)

### 

Data collection: *CrysAlis Pro* (Oxford Diffraction, 2009 ▶); cell refinement: *CrysAlis Pro*; data reduction: *CrysAlis Pro*; program(s) used to solve structure: *SHELXS97* (Sheldrick, 2008 ▶); program(s) used to refine structure: *SHELXL97* (Sheldrick, 2008 ▶); molecular graphics: *XP* (Siemens, 1994 ▶); software used to prepare material for publication: *SHELXL97*.

## Supplementary Material

Crystal structure: contains datablocks I, global. DOI: 10.1107/S1600536809030815/at2855sup1.cif

Structure factors: contains datablocks I. DOI: 10.1107/S1600536809030815/at2855Isup2.hkl

Additional supplementary materials:  crystallographic information; 3D view; checkCIF report

## Figures and Tables

**Table 1:** Hydrogen-bond geometry (Å, °)

*D*—H⋯*A*	*D*—H	H⋯*A*	*D*⋯*A*	*D*—H⋯*A*
N1—H01⋯O3^i^	0.842 (16)	2.027 (16)	2.8465 (14)	164.1 (14)
C8—H8⋯O2^i^	0.95	2.51	2.9686 (16)	110
C11—H11*B*⋯O2^ii^	0.98	2.52	3.457 (2)	160
C3—H3*B*⋯O3^iii^	0.98	2.67	3.5693 (17)	152
C1—H1*C*⋯S^iv^	0.98	2.95	3.9281 (16)	177

Symmetry codes: (i) 
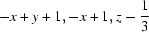
; (ii) 
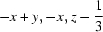
; (iii) 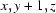; (iv) 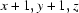.

**Fig. 1.**
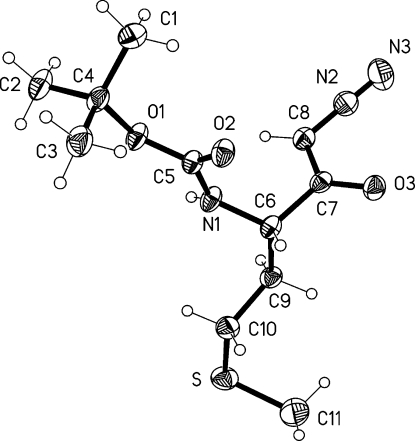
The molecule of the title compound in the crystal. Ellipsoids correspond to 50% probability levels.

**Fig. 2.**
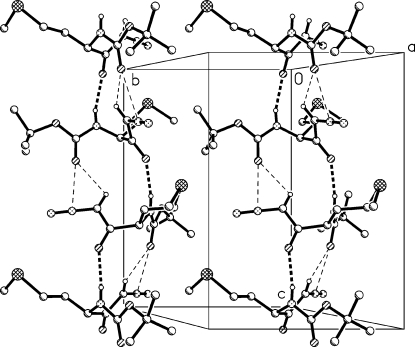
Packing diagram of the title compound viewed perpendicular to the yz plane. Classical H bonds are represented by thick dashed lines, and the three-centre interaction (see text) by thin dashed lines. H atoms not involved in H bonding are omitted for clarity.

## supplementary crystallographic information

### Comment

α-Diazocarbonyl compounds find widespread applications in organic and, especially, natural product synthesis (Padwa & Weingarten, 1996). The ready availability, relative stability and facile decomposition of these compounds under various conditions (*e.g.* thermal, photochemical; acid-, base- and transition-metal-catalysis) make them useful intermediates (Doyle *et al.* 1998). Furthermore, α-diazoketones undergo a variety of transformations such as cyclopropanation, aziridine formation, ylide formation, C–H or C–*X* insertion reactions and cyclization reactions (Ye & McKervey, 1994). These reactions are chemoselective, and promote the formation of new carbon-carbon and carbon-heteroatom bonds under mild conditions. Asymmetric versions of diazocarbonyl reactions have been reported to produce enantiomerically pure compounds (Doyle & McKervey, 1997). One such method is the Arndt-Eistert synthesis, which consists of conversion of activated carboxylic acids to diazoketones by the action of diazomethane, followed by Wolf rearrangement. The method has become widely used in recent years for the synthesis of β-peptides and β-amino acid derivatives from appropriately protected α-amino acids (Müller *et al.* 1998). Here we present the structure of an α-diazocarbonyl compound based on methionine.

The structure of the title compound is shown in Fig. 1. Molecular dimensions may be regarded as normal. The two essentially planar groupings N1,O1,O2,C2,4,5,6 and N2,N3,O3,C6,7,8 (r.m.s. deviations 0.04, 0.02 Å) subtend an interplanar angle of 84.75 (3)°. The atom chain C2 to C6 displays an extended conformation (minimum absolute torsion angle 170°).

The main feature of the molecular packing is the classical H bond N1—H1···O3, which links the molecules *via* the 3_1_ screw operator to form chains parallel to the *z* axis (Fig. 2). Within the chains, an unusual three-centre interaction is also observed, whereby the carbonyl oxygen O2 is involved in short contacts to H8 and N2 of the diazocarbonyl group of a neighbouring molecule. The former is far from linear (angle 110°) but this is not unusual for three-centre interactions. The latter may be interpreted as a dipole-dipole interaction [dimensions: N2···O2 2.8141 (14) Å, C8—N2···O2 73.5 (1)°]. The remaining "weak" C—H···O interactions (Table 1) link neighbouring chains; H3B···O3 is implicit between the chains of Fig. 2 but is omitted for clarity.

### Experimental

10 mmol of BOC-protected methionine was dissolved in 50 ml of dry distilled THF under inert conditions. To maintain basic conditions 12 mmol (1.66 ml) of triethylamine was added. Then 10 mmol (0.95 ml) of ethyl chloroformate was added, and the mixture stirred for 15 min at 248 K. 13 mmol of diazomethane were then added at 268 K and the mixture was further stirred for 30 min. After this temperature was allowed to rise to room temperature over 3 h. The reaction was then quenched with 3–4 drops of glacial acetic acid. The solvent was evaporated under vacuum. The residue was dissolved in ethyl acetate, extracted with aq. solutions of NaHCO_3_ and NH_4_Cl and dried over anhydrous MgSO_4_. The crude product was purified by column chromatography (yield 85%; m.p.326-328 K).

### Refinement

The NH hydrogen was refined freely. Methyl H atoms were identified in difference syntheses, idealized and refined as rigid groups with C—H 0.98 Å and H—C—H angles 109.5°, allowed to rotate but not tip. Other H atoms were placed in calculated positions and refined using a riding model with C—H 0.98 Å (methylene) or 0.99 Å (methine); hydrogen *U* values were fixed at 1.5 × *U*(eq) of the parent atom for methyl H and 1.2 × *U*(eq) of the parent atom for other C—H. Data are 100% complete to 2θ 145°. The absolute configuration *S* at C6 (and thus the space group *P*3_1_ rather than its enantiomer *P*3_2_) was determined by the Flack (1983) parameter, which refined to 0.023 (10).

### Crystal data

**Table:**

C_11_H_19_N_3_O_3_S	*D*_x_ = 1.183 Mg m^−^^3^
*M**_r_* = 273.35	Cu *K*α radiation, λ = 1.54184 Å
Trigonal, *P*3_1_	Cell parameters from 14422 reflections
Hall symbol: P 31	θ = 3.2–75.7°
*a* = 9.7915 (3) Å	µ = 1.93 mm^−^^1^
*c* = 13.8581 (5) Å	*T* = 100 K
*V* = 1150.62 (6) Å^3^	Tablet, colourless
*Z* = 3	0.20 × 0.20 × 0.15 mm
*F*(000) = 438	

### Data collection

**Table:**

Oxford Diffraction Xcalibur Nova A diffractometer	3073 independent reflections
Radiation source: Nova (Cu) X-ray Source	3051 reflections with *I* > 2σ(*I*)
mirror	*R*_int_ = 0.032
Detector resolution: 10.3543 pixels mm^-1^	θ_max_ = 75.6°, θ_min_ = 5.2°
ω scans	*h* = −12→12
Absorption correction: multi-scan (*CrysAlis PRO*; Oxford Diffraction, 2009)	*k* = −12→12
*T*_min_ = 0.717, *T*_max_ = 1.000	*l* = −17→15
16152 measured reflections	

### Refinement

**Table:**

Refinement on *F*^2^	Secondary atom site location: difference Fourier map
Least-squares matrix: full	Hydrogen site location: inferred from neighbouring sites
*R*[*F*^2^ > 2σ(*F*^2^)] = 0.023	H atoms treated by a mixture of independent and constrained refinement
*wR*(*F*^2^) = 0.060	*w* = 1/[σ^2^(*F*_o_^2^) + (0.0342*P*)^2^ + 0.1663*P*] where *P* = (*F*_o_^2^ + 2*F*_c_^2^)/3
*S* = 1.05	(Δ/σ)_max_ < 0.001
3073 reflections	Δρ_max_ = 0.15 e Å^−^^3^
171 parameters	Δρ_min_ = −0.14 e Å^−^^3^
1 restraint	Absolute structure: Flack (1983), 1474 Freidel pairs
Primary atom site location: structure-invariant direct methods	Flack parameter: 0.023 (10)

### Special details

**Table:**

Geometry. All e.s.d.'s (except the e.s.d. in the dihedral angle between two l.s. planes) are estimated using the full covariance matrix. The cell e.s.d.'s are taken into account individually in the estimation of e.s.d.'s in distances, angles and torsion angles; correlations between e.s.d.'s in cell parameters are only used when they are defined by crystal symmetry. An approximate (isotropic) treatment of cell e.s.d.'s is used for estimating e.s.d.'s involving l.s. planes.Short contact: 2.8141 (14) N2 - O2_$1; 73.5 (1) C8 - N2 - O2_$1; Operator $1 - *x* + *y*+1, -*x* + 1, *z* - 1/3
Refinement. Refinement of *F*^2^ against ALL reflections. The weighted *R*-factor *wR* and goodness of fit *S* are based on *F*^2^, conventional *R*-factors *R* are based on *F*, with *F* set to zero for negative *F*^2^. The threshold expression of *F*^2^ > σ(*F*^2^) is used only for calculating *R*-factors(gt) *etc*. and is not relevant to the choice of reflections for refinement. *R*-factors based on *F*^2^ are statistically about twice as large as those based on *F*, and *R*- factors based on ALL data will be even larger.

### Fractional atomic coordinates and isotropic or equivalent isotropic displacement parameters (Å^2^)

**Table:**

	*x*	*y*	*z*	*U*_iso_*/*U*_eq_	
S	0.08692 (4)	−0.10432 (4)	0.14682 (2)	0.03648 (9)	
O1	0.57182 (11)	0.62115 (10)	0.24352 (6)	0.02868 (19)	
O2	0.57776 (11)	0.51506 (10)	0.38885 (6)	0.02855 (19)	
O3	0.66256 (11)	0.16891 (11)	0.38136 (6)	0.02857 (19)	
N1	0.49938 (13)	0.36907 (12)	0.25188 (8)	0.0259 (2)	
H01	0.4999 (17)	0.3757 (17)	0.1913 (12)	0.021 (3)*	
N2	0.88980 (14)	0.31145 (15)	0.24685 (9)	0.0340 (3)	
N3	1.00277 (17)	0.3112 (2)	0.25596 (10)	0.0492 (4)	
C1	0.76526 (17)	0.85794 (16)	0.33133 (11)	0.0350 (3)	
H1A	0.7632	0.7991	0.3890	0.052*	
H1B	0.7907	0.9647	0.3499	0.052*	
H1C	0.8455	0.8640	0.2865	0.052*	
C2	0.60734 (19)	0.86229 (16)	0.19163 (11)	0.0369 (3)	
H2A	0.6887	0.8685	0.1477	0.055*	
H2B	0.6307	0.9690	0.2085	0.055*	
H2C	0.5042	0.8059	0.1600	0.055*	
C3	0.47294 (18)	0.75163 (17)	0.35029 (12)	0.0364 (3)	
H3A	0.3714	0.6914	0.3172	0.055*	
H3B	0.4883	0.8548	0.3693	0.055*	
H3C	0.4739	0.6940	0.4079	0.055*	
C4	0.60502 (15)	0.77449 (14)	0.28283 (10)	0.0278 (3)	
C5	0.55262 (14)	0.50412 (13)	0.30286 (9)	0.0244 (2)	
C6	0.48307 (14)	0.22893 (14)	0.29774 (9)	0.0240 (2)	
H6	0.4329	0.2173	0.3624	0.029*	
C7	0.64131 (14)	0.23513 (13)	0.31215 (9)	0.0234 (2)	
C8	0.75513 (15)	0.31186 (15)	0.23793 (10)	0.0288 (3)	
H8	0.7356	0.3609	0.1847	0.035*	
C9	0.37572 (14)	0.08310 (13)	0.23693 (9)	0.0257 (2)	
H9A	0.4220	0.0957	0.1718	0.031*	
H9B	0.3707	−0.0110	0.2670	0.031*	
C10	0.20871 (15)	0.05652 (15)	0.22743 (10)	0.0300 (3)	
H10A	0.2144	0.1544	0.2032	0.036*	
H10B	0.1588	0.0339	0.2920	0.036*	
C11	0.0623 (2)	−0.26955 (18)	0.21754 (14)	0.0502 (4)	
H11A	0.1660	−0.2559	0.2331	0.075*	
H11B	0.0012	−0.3671	0.1806	0.075*	
H11C	0.0062	−0.2755	0.2774	0.075*	

### Atomic displacement parameters (Å^2^)

**Table:**

	*U*^11^	*U*^22^	*U*^33^	*U*^12^	*U*^13^	*U*^23^
S	0.03136 (16)	0.03576 (17)	0.03360 (17)	0.01025 (14)	−0.00604 (13)	−0.00369 (14)
O1	0.0426 (5)	0.0199 (4)	0.0260 (4)	0.0176 (4)	−0.0038 (4)	−0.0017 (3)
O2	0.0390 (5)	0.0245 (4)	0.0246 (5)	0.0177 (4)	−0.0050 (3)	−0.0027 (3)
O3	0.0319 (4)	0.0295 (4)	0.0243 (4)	0.0153 (4)	0.0004 (3)	0.0041 (3)
N1	0.0384 (6)	0.0196 (5)	0.0208 (5)	0.0154 (4)	−0.0023 (4)	−0.0016 (4)
N2	0.0324 (6)	0.0379 (6)	0.0292 (6)	0.0157 (5)	0.0058 (4)	0.0081 (4)
N3	0.0392 (7)	0.0717 (10)	0.0426 (8)	0.0320 (7)	0.0095 (6)	0.0198 (7)
C1	0.0336 (7)	0.0248 (6)	0.0424 (8)	0.0115 (5)	−0.0030 (6)	−0.0028 (5)
C2	0.0521 (8)	0.0249 (6)	0.0381 (8)	0.0224 (6)	−0.0028 (6)	0.0013 (5)
C3	0.0400 (8)	0.0309 (7)	0.0456 (8)	0.0230 (6)	0.0044 (6)	0.0018 (6)
C4	0.0334 (6)	0.0184 (5)	0.0335 (7)	0.0145 (5)	−0.0017 (5)	−0.0019 (5)
C5	0.0271 (6)	0.0203 (5)	0.0282 (6)	0.0137 (5)	−0.0001 (4)	−0.0001 (4)
C6	0.0293 (6)	0.0198 (5)	0.0239 (6)	0.0131 (5)	0.0010 (4)	0.0005 (4)
C7	0.0275 (6)	0.0170 (5)	0.0228 (6)	0.0091 (4)	−0.0009 (4)	−0.0021 (4)
C8	0.0289 (6)	0.0277 (6)	0.0289 (6)	0.0135 (5)	0.0024 (5)	0.0051 (5)
C9	0.0273 (6)	0.0199 (5)	0.0289 (6)	0.0110 (5)	−0.0003 (5)	−0.0015 (4)
C10	0.0274 (6)	0.0261 (6)	0.0356 (7)	0.0126 (5)	−0.0017 (5)	−0.0014 (5)
C11	0.0479 (9)	0.0284 (7)	0.0653 (11)	0.0124 (7)	−0.0165 (8)	−0.0071 (7)

### Geometric parameters (Å, °)

**Table:**

S—C11	1.8015 (17)	C1—H1A	0.9800
S—C10	1.8089 (13)	C1—H1B	0.9800
O1—C5	1.3450 (14)	C1—H1C	0.9800
O1—C4	1.4727 (14)	C2—H2A	0.9800
O2—C5	1.2107 (16)	C2—H2B	0.9800
O3—C7	1.2323 (15)	C2—H2C	0.9800
N1—C5	1.3528 (15)	C3—H3A	0.9800
N1—C6	1.4468 (15)	C3—H3B	0.9800
N2—N3	1.1145 (18)	C3—H3C	0.9800
N2—C8	1.3264 (18)	C6—H6	1.0000
C1—C4	1.5163 (19)	C8—H8	0.9500
C2—C4	1.5223 (19)	C9—H9A	0.9900
C3—C4	1.5189 (19)	C9—H9B	0.9900
C6—C7	1.5330 (17)	C10—H10A	0.9900
C6—C9	1.5340 (16)	C10—H10B	0.9900
C7—C8	1.4237 (17)	C11—H11A	0.9800
C9—C10	1.5276 (17)	C11—H11B	0.9800
N1—H01	0.842 (16)	C11—H11C	0.9800
			
C11—S—C10	100.36 (7)	H2A—C2—H2B	109.5
C5—O1—C4	120.53 (10)	C4—C2—H2C	109.5
C5—N1—C6	120.23 (11)	H2A—C2—H2C	109.5
N3—N2—C8	178.84 (15)	H2B—C2—H2C	109.5
O1—C4—C1	110.78 (10)	C4—C3—H3A	109.5
O1—C4—C3	109.87 (10)	C4—C3—H3B	109.5
C1—C4—C3	112.46 (12)	H3A—C3—H3B	109.5
O1—C4—C2	101.65 (10)	C4—C3—H3C	109.5
C1—C4—C2	110.15 (11)	H3A—C3—H3C	109.5
C3—C4—C2	111.43 (11)	H3B—C3—H3C	109.5
O2—C5—O1	126.21 (10)	N1—C6—H6	108.5
O2—C5—N1	124.23 (11)	C7—C6—H6	108.5
O1—C5—N1	109.57 (11)	C9—C6—H6	108.5
N1—C6—C7	112.93 (10)	N2—C8—H8	121.7
N1—C6—C9	109.93 (10)	C7—C8—H8	121.7
C7—C6—C9	108.48 (9)	C10—C9—H9A	109.1
O3—C7—C8	123.12 (12)	C6—C9—H9A	109.1
O3—C7—C6	120.91 (11)	C10—C9—H9B	109.1
C8—C7—C6	115.84 (11)	C6—C9—H9B	109.1
N2—C8—C7	116.62 (12)	H9A—C9—H9B	107.8
C10—C9—C6	112.51 (10)	C9—C10—H10A	109.1
C9—C10—S	112.65 (9)	S—C10—H10A	109.1
C5—N1—H01	117.4 (10)	C9—C10—H10B	109.1
C6—N1—H01	120.3 (10)	S—C10—H10B	109.1
C4—C1—H1A	109.5	H10A—C10—H10B	107.8
C4—C1—H1B	109.5	S—C11—H11A	109.5
H1A—C1—H1B	109.5	S—C11—H11B	109.5
C4—C1—H1C	109.5	H11A—C11—H11B	109.5
H1A—C1—H1C	109.5	S—C11—H11C	109.5
H1B—C1—H1C	109.5	H11A—C11—H11C	109.5
C4—C2—H2A	109.5	H11B—C11—H11C	109.5
C4—C2—H2B	109.5		
			
C5—O1—C4—C1	66.13 (15)	C9—C6—C7—O3	−89.46 (13)
C5—O1—C4—C3	−58.74 (14)	N1—C6—C7—C8	−35.64 (15)
C5—O1—C4—C2	−176.85 (11)	C9—C6—C7—C8	86.47 (12)
C4—O1—C5—O2	−9.28 (19)	O3—C7—C8—N2	−0.93 (19)
C4—O1—C5—N1	170.45 (10)	C6—C7—C8—N2	−176.76 (11)
C6—N1—C5—O2	−5.96 (19)	N1—C6—C9—C10	−62.16 (13)
C6—N1—C5—O1	174.30 (10)	C7—C6—C9—C10	173.92 (10)
C5—N1—C6—C7	−76.13 (14)	C6—C9—C10—S	174.67 (9)
C5—N1—C6—C9	162.57 (10)	C11—S—C10—C9	69.91 (11)
N1—C6—C7—O3	148.43 (11)		

### Hydrogen-bond geometry (Å, °)

**Table:**

*D*—H···*A*	*D*—H	H···*A*	*D*···*A*	*D*—H···*A*
N1—H01···O3^i^	0.842 (16)	2.027 (16)	2.8465 (14)	164.1 (14)
C8—H8···O2^i^	0.95	2.51	2.9686 (16)	110
C11—H11B···O2^ii^	0.98	2.52	3.457 (2)	160
C3—H3B···O3^iii^	0.98	2.67	3.5693 (17)	152
C1—H1C···S^iv^	0.98	2.95	3.9281 (16)	177

Symmetry codes: (i) −*x*+*y*+1, −*x*+1, *z*−1/3; (ii) −*x*+*y*, −*x*, *z*−1/3; (iii) *x*, *y*+1, *z*; (iv) *x*+1, *y*+1, *z*.
